# Effect of Neutrophil to Lymphocyte ratio on antidepressant treatment response: moderating effect of sex and mediating effect of Hippocampal volumes.

**DOI:** 10.1192/j.eurpsy.2023.564

**Published:** 2023-07-19

**Authors:** M. Paolini, Y. Harrington, J. Ernst, R. Zanardi, S. Poletti, F. Benedetti

**Affiliations:** ^1^Vita-Salute San Raffaele University Milan, Italy, Milano; ^2^IRCCS San Raffaele Scientific Institute, Milan, Italy, Milano

## Abstract

**Introduction:**

In recent years much focus has been put on the role of immune/inflammatory alterations in affecting Major Depression (MDD) development and antidepressant efficacy. Neutrophil-to-lymphocyte ratio (NLR) is an inexpensive inflammatory marker shown to be elevated in depressed patients, with large population studies reporting this effect only in women. However, its relation to treatment response is much less clear. Reduced hippocampal volumes (HV) are among the few consistent brain structural predictors of poor treatment response, and they have been shown to be influenced by inflammatory status.

**Objectives:**

To investigate the effect of NLR on treatment response in MDD patients, testing a possible moderating role of sex. To investigate the effect of NLR on HV and test a possible mediating role of the latter in the relation between NLR and treatment response.

**Methods:**

Our study was performed on a sample of 120 MDD inpatients suffering from a non psychotic depressive episode (F=78; M=42). Depression severity was assessed via the Hamilton Depression Rating Scale (HDRS), both at admission and discharge; as a measure of treatment response, delta HDRS was calculated subtracting the two scores. NLR was calculated for each subject. Patients underwent 3T MRI acquisition and bilateral HV were estimated.

**Results:**

We found a significant moderating effect of sex on the relationship between NLR and Delta HDRS (p < 0.001): a negative relation was found in women (p < 0.001) and a positive one in men (p = 0.042). NLR was found to negatively affect left HV in the whole sample (p = 0.027) and in women (p = 0.038). A positive effect on Delta HDRS was found for both left (p = 0.038) and right (p = 0.027) HV. Finally, we found a significant indirect effect of NLR values on Delta HDRS through left HV in women (95% BCa CI [- 0.948, -0.017]); the direct effect of NLR on Delta HDRS also remained significant (p = 0.002).

**Conclusions:**

Sex was found to moderate the relation between NLR and treatment response. The detrimental effect in women is in line with previous reports linking inflammation to hampered antidepressant effect; the positive one in men is more surprising: however, the only studies to date on the effect of NLR on antidepressant efficacy report a positive effect in patients with psychotic depression. In women we found NLR to affect treatment response partially through its effect on left HV, providing a possible, albeit incomplete, mechanistic explanation of the effect of inflammatory status on antidepressant efficacy.

**Disclosure of Interest:**

None Declared

